# Inpatient charges among HIV/AIDS patients in Rhode Island from 2000–2004

**DOI:** 10.1186/1472-6963-9-3

**Published:** 2009-01-07

**Authors:** Kamil E Barbour, Anthony Fabio, Deborah N Pearlman

**Affiliations:** 1 Brown University Program in Public Health, Center for Population Health and Clinical Epidemiology, 121 South Main Street, Providence, RI 02912, USA; 2 University of Pittsburgh, Center for Injury Research and Control, 3471 5th Ave # 810, Pittsburgh, PA 15213, USA; 3 University of Pittsburgh, Center for Aging and Population Health, 130 N Bellefield Avenue, Pittsburgh, PA, 15213, USA

## Abstract

**Background:**

Inpatient HIV/AIDS charges decreased from 1996–2000. This decrease was mainly attributable to treatment of HIV/AIDS patients with Highly Active Antiretroviral Therapy (HAART). This study aims to evaluate the trend in inpatient charges from 2000–2004.

**Methods:**

Rhode Island Hospital Discharge Data (HDD) from 2000 to 2004 was used. International Classification of Disease (ICD-9) diagnosis code 042–044 was used to identify HIV/AIDS admissions. The final study population included 1927 HIV/AIDS discharges. We used a multivariable linear regression model to examine the factors associated with inflation adjusted inpatient charges.

**Results:**

We found a significant increase in inpatient charges from 2000–2004 after adjusting for length of stay (LOS), gender, age, race and point of entry for hospitalization. In addition to calendar year, LOS, gender and race were also associated with inpatient charges.

**Conclusion:**

HIV/AIDS inpatient charges increased after adjusting for inflation despite earlier studies that showed a decline. Our results have implications for uninsured, as well as insured HIV/AIDS patients who do not have a medical plan that covers their charges sufficiently. Future research should investigate what factors are contributing to rising inpatient charges among HIV/AIDS patients.

## Background

Recent statistics show that an estimated 623,435 individuals in the United States (US) are living with HIV[1]. HIV/AIDS continues to be a major financial burden on the health care system in the US[2]. The average annual HIV/AIDS charges per patient were estimated to be approximately $18,000 from 1996–2000. Studies estimate that around 50% of these average annual charges were attributable to inpatient care [2-4]. Inpatient charges for HIV/AIDS patients are comparable to charges from other chronic diseases. Reed et al. estimated that average annual inpatient charges for intracerebral hemorrhage (ICH) were $10,241[5]. The majority of charges for health services among individuals infected with HIV/AIDS come from public sources such as Medicaid, Ryan White, and Medicare, making it imperative for those who allocate health care dollars for HIV treatment to be aware of the cost of treating people with HIV/AIDS [6]. In 1996, combination antiretroviral drug therapy became more common[7]. The average annual number of admissions and charges for inpatient and overall HIV/AIDS care decreased between 1996–2000 [8,9]. This decrease was mainly attributable to Highly Active Antiretroviral Therapy (HAART), which enabled many HIV/AIDS patients to be treated on an outpatient basis, leading to an overall decrease in inpatient length of stay (LOS) and inpatient HIV/AIDS charges per person [9,10]. This decline in HIV/AIDS charges was in sharp contrast to the increase in charges that Hellinger had forecasted from 1989–1995 [11-13]. However, these forecasts were made prior to the advent of HAART. Studies suggest that an estimated 20–30% of HIV/AIDS patients are uninsured [14,15] making it essential to continue to examine the burden of annual HIV/AIDS charges to determine what is impacting HIV/AIDS charges and how to decrease these charges.

The purpose of his study was to describe trends in inpatient charges in Rhode Island between 2000 and 2004 for hospital discharges where HIV/AIDS was the primary reason for admission. In addition, socioeconomic demographic and clinical variations in inpatient hospital charges among these discharges were examined.

## Methods

### Study Population

The Rhode Island Department of Health's Center for Health Data and Analysis collects Hospital Discharge Data (HDD) each year on inpatients from all 13 licensed acute care hospitals and the rehabilitation facility in Rhode Island from 2000–2004. The data reported to the Department of Health includes information on demographic data, clinical items, and hospital charges[16]. The data were restricted to the years 2000 to 2004, to discharges with specific ICD 9 codes, and to a patient population aged 18+. Because the file did not include a unique medical identifier, it was not possible to distinguish between patients with a single admission during the study period versus multiple admissions. The final study population included 1927 HIV/AIDS discharges. International Classification of Disease (ICD-9) diagnosis code 042–044 was used to identify HIV/AIDS admissions. We decided to include diagnoses that were not primary (> 50%) based on the need to decrease the large amount of false negatives that would result by excluding secondary and tertiary diagnoses of HIV/AIDS. The Rhode Island HDD is a public use file. We obtained consent to use this data from the Rhode Island Department of Health.

### Outcome Measure

Inpatient charges (in dollars) were measured as the total charges of an admission to the hospital. Inpatient charges do not include professional fees, and non-covered charges. Non-covered charges are charges that patients or insurance providers are not required to pay. Emergency department charges incurred prior to admission are included in total charges. Inpatient charges were adjusted for inflation. Charges for subsequent years were deflated to 2000 dollars. This was done by taking into account the average annual percent growth in National Health Expenditures. This measure estimates the amount spent on all health services, supplies, health-related research, and construction activities in the United States during the calendar year.

### Covariates

The independent variables included, LOS, and point of entry for hospitalization. LOS was measured in days. Point of entry for hospitalization had two categories: Emergency Room, and patients who were referred to the hospital through a physician, hospital clinic referral, or a facility referral. Specific data on income, education, and other components of SES were not available in the HDD data. Therefore, we utilized the point of entry for hospitalization variable as a proxy for socioeconomic status (SES). Previous studies have shown that patients referred through the emergency room are of lower SES when compared to patients with other types of hospital referrals (physician, clinic, or facility) [17,18]. The demographic variables included in this study were age, race, and gender. Age was a continuous variable that was measured in years. Race category consisted of six categories: white, black, Hispanic, Asian, unknown and other.

### Statistical Analysis

Rhode Island HDD data from 2000–2004 were analyzed using the Statistical Analysis System (SAS, version 9.1; SAS Institute, Cary, NC). We performed a descriptive analysis of demographic and clinical characteristics by year, using one-way Analysis of Variance (ANOVA) and two-sample t-tests for continuous outcomes and Pearson's chi-square test of independence or Fisher's exact test for categorical outcomes. Fisher's exact test replaced Pearson's chi-square test when the expected cell count was less than 5 for a particular descriptive analysis. We used histograms and the Kolmogorov-Smirnov test to determine if inpatient charges and LOS followed a normal distribution. Due to the skewness of the data a natural log transformation was taken for LOS and inpatient charges. ANOVAs and t-tests were performed on the log transformed data which was subsequently back-transformed to generate the geometric means and standard errors for LOS and inpatient charges. Geometric means and standard deviations reported are likely to be smaller than normal calculations involving untransformed data. The reason for this is that the natural log transformation reduces the influence of extreme observations and outliers resulting in exponentiated geometric values that inherit the reduced influence of extreme observations. We used 2 multivariable linear regression models to compare the coefficients for different years across the models to understand how they change when LOS is controlled. The 2 models examined factors associated with inflation adjusted inpatient charges, with LOS excluded from the first model and included in the second model. Comparing the 2 models enabled us to examine how LOS mediates the effects of sociodemographic and clinical variables. The inflation adjusted inpatient charges were used as the outcome variable. The other independent variables in these models included year, age, race, gender, and point of entry for hospitalization. To meet the assumption of normality of the linear regression model, we performed a natural log transformation for inpatient charges and LOS. Regression coefficients were then exponentiated to obtain the estimated mean percentage increase in inpatient charges for each independent variable. We used a test of linear trend to examine if certain factors are related to each other linearly. All tests were two-sided with a type I error (α) of 0.05. The Bonferroni adjustment of α/k (k number of comparisons) for post-hoc ANOVA analysis was used to address the multiple comparisons problem.

## Results

Among HIV/AIDS discharges with a primary diagnosis in Rhode Island from 2000–2004, 66.6% were male, 44.5% were white, 36.8% were black and 13.5% were Hispanic. The mean age of patients was approximately 42.7 ± 9.0 years. An estimated three quarters of the patients were admitted through the emergency room and the geometric mean for LOS was 4.7 ± 2.4 days. The inflation adjusted geometric mean inpatient charges over the 5 year time period was $11,480 ± 2.9.

### Univariate Analyses

Inflation adjusted inpatient charges differed by point of entry for hospitalization in 2001, and gender from 2002–2004 (Table 1). In 2001, patients that were admitted through an emergency room ($9,760 ± 3.9) had significantly higher inpatient charges than patients who were admitted by physician, clinic, or facility referral ($7,663 ± 2.6). Males had significantly higher inpatient charges than women in 2002 ($12,544 ± 2.8 versus $9,404 ± 2.4), 2003 ($16,652 ± 2.7 versus $14,561 ± 2.4), and 2004 ($18,966 ± 3.0 versus $14,815 ± 2.7).

**Table 1:** Inpatient Charges among HIV/AIDS patients in Rhode Island per year, 2000–2004.

	**2000 (N = 402)** **Mean ± SE**	**2001 (N = 396)** **Mean ± SE**	**2002 (N = 343)** **Mean ± SE**	**2003 (N = 416)** **Mean ± SE**	**2004 (N = 370)** **Mean ± SE**
Gender					
Male	8708 ± 1.06	8732 ± 1.1	12544* ± 1.07	16652* ± 1.07	18966* ± 1.07
Female	8641 ± 1.09	10044 ± 1.06	9404 ± 1.09	14561 ± 1.08	14815 ± 1.09
Race					
Hispanic	7859 ± 1.14	9710 ± 1.18	11452 ± 1.13	10217 ± 1.16	18391 ± 1.15
Black	8869 ± 1.09	9254 ± 1.09	9943 ± 1.09	13162 ± 1.08	14197 ± 1.10
White	8901 ± 1.08	9121 ± 1.08	12729 ± 1.09	13307 ± 1.08	18035 ± 1.09
Asian	8698 ± 2.02	9142 ± 1.70	14600 ± 1.45	N/A	N/A
Other	8435 ± 1.24	11129 ± 1.42	14379 ± 1.31	9055 ± 1.46	28529 ± 1.48
Unknown	6557 ± 1.77	2796 ± 1.83	N/A	10715 ± 1.51	20758 ± 1.34
Point of entry for Hospitalization					
PCF^a^	8104 ± 1.09	7663* ± 1.10	12068 ± 1.12	13837 ± 1.2	17793 ± 1.12
ER^b^	8900 ± 1.06	9760 ± 1.06	11231 ± 1.06	12530 ± 1.06	17204 ± 1.07

Note: Geometric means and standard errors are presented in the table.

^a ^Physician, Clinical, or Facility.

^b ^Emergency Room.

*P < 0.05

†Point of entry for hospitalization is missing 1 observation in 2003.

N/A: Not applicable if there are no observations.

The test of trend showed a significantly increasing trend for inpatient charges among HIV/AIDS admissions from 2000–2004 (Table 2). Average charges per HIV/AIDS admission was least in 2000 ($8,771 ± 4.6) and greatest in 2004 ($17,367 ± 2.9). The greatest one-year increase in charges (35.6%) and LOS (12.8%) occurred from 2003–2004. LOS was found to be significantly higher in 2004 compared to 2001 (p = 0.001), after adjusting for multiple comparisons by using a Bonferroni adjustment. Age, race, LOS and inpatient charges differed significantly by year. However, the test of trend showed that LOS did not have a significantly increasing linear trend from 2000–2004.

**Table 2:** Demographic and Clinical Characteristics of HIV/AIDS patients by Year in Rhode Island, 2000–2004

	**2000** **N (%)** **Mean ± SE**	**2001** **N (%)** **Mean ± SE**	**2002** **N (%)** **Mean ± SE**	**2003** **N (%)** **Mean ± SE**	**2004** **N (%)** **Mean ± SE**
Inpatient Charges†,*	8771 ± 1.05	9182 ± 1.05	11428 ± 1.06	12802 ± 1.05	17367 ± 1.05
LOS*	4.6 ± 1.05	4.3 ± 1.05	4.7 ± 1.05	4.7 ± 1.04	5.3 ± 1.05
Gender					
Male	264 (65.7)	285 (72.0)	232 (67.6)	267 (64.2)	236 (63.8)
Female	138 (34.3)	111 (28.0)	111 (32.4)	149 (35.8)	134 (36.2)
Age*	42.9 ± 1.57	41.7 ± 1.57	42.9 ± 1.63	42.9 ± 1.56	43.2 ± 1.60
Race*					
Hispanic	58 (14.4)	39 (9.8)	63 (18.4)	44 (16.9)	57 (15.4)
Black	133 (33.1)	134 (33.8)	139 (40.5)	175 (42.1)	129 (34.9)
White	185 (46.0)	207 (52.3)	120 (35.0)	183 (44.0)	163 (44.0)
Asian	2 (0.5)	4 (1.0)	7 (2.0)	1 (0.2)	1 (0.3)
Other	21 (5.2)	9 (2.3)	13 (3.8)	7 (1.7)	7 (1.9)
Unknown	3 (0.8)	3 (0.8)	1 (0.3)	6 (1.4)	13 (3.5)
Point of entry for Hospitalization					
PCF^a^	105 (26.1)	100 (25.2)	83 (24.2)	99 (23.9)	97 (26.2)
ER^b^	297 (73.9)	296 (74.8)	260 (75.8)	316 (76.1)	273 (73.8)

Note: Geometric means and standard errors are presented in the table.

^a ^Physician, Clinical, or Facility.

^b ^Emergency Room.

*P < 0.05

†P for linear trend < 0.05.

‡Point of entry for hospitalization is missing 1 observation in 2003.

### Multivariable Analyses

In both multivariable models inpatient charges significantly increased over time based on the test of trend after adjusting for LOS, calendar year gender age, race and point of entry for hospitalization (Table 3, 4). In the first model that excluded LOS, inflation adjusted log inpatient charges were significantly higher in 2002 (31%), 2003 (49%), and 2004 (101%) compared to 2000. Males had a 17% (95% CI, 6–29) significant mean increase in inpatient charges than females. This model (R^2 ^= 0.0646) accounted for only 6.46% of the variance in log inpatient charges. This small R^2 ^is primarily due to not having LOS in the model.

**Table 3:** Relationship of Demographic and Clinical Characteristics to Inpatient Charges among HIV/AIDS Patients in Rhode Island excluding LOS, 2000–2004.

	**Estimated Increase in Inpatient Charges Adjusted %**	**95% CI**
Year†		
2000	--	
2001	3	-10 to 19
2002*	31	13 to 52
2003*	49	29 to 71
2004*	101	74 to 133
Age^a^	-0.5	-1 to 0
Gender		
Female	--	
Male*	17	6 to 29
Race		
White	--	
Black	-8	-17 to 2
Hispanic	16	-32 to 95
Asian	-6	-19 to 8
Other	27	-4 to 67
Unknown	-6	-38 to 41
Point of entry for Hospitalization		
PCF^b^	--	
ER^c^	3	-12 to 25

^a ^Age in 1 year increments, are modeled as continuous covariates.

^b ^Physician, Clinical, or Facility.

^c ^Emergency Room.

*P < 0.05

†P for linear trend < 0.05.

**Table 4:** Relationship of Demographic and Clinical Characteristics to Inpatient Costs among HIV/AIDS Patients in Rhode Island, 2000–2004

	**Estimated Increase in Inpatient Costs Adjusted %**	**95% CI**
Year†		
2000	--	
2001*	11	3.5 to 20.1
2002*	31	21.3 to 41.6
2003*	45	35.0 to 56.3
2004*	73	60.7 to 87.0
LOS^a^, *	166	159.0 to 173.2
Age^a^	-2	-4.5 to 0.8
Gender		
Female	--	
Male*	10	4.6 to 15.9
Race		
White	--	
Black*	-11	-16.8 to -6.4
Hispanic	-17	-37.2 to 8.4
Asian*	-11	-17.8 to -4.5
Other*	19	3.3 to 37.5
Unknown	7	-28.3 to 9.8
Point of entry for Hospitalization		
PCF^b^	--	
ER^c^	2	-0.4 to 7.4

^a ^LOS in 1 day increments, Age in 1 year increments, are modeled as continuous covariates.

^b ^Physician, Clinical, or Facility

^c ^Emergency Room

*P < 0.05

†P for linear trend < 0.05

In the model that included LOS, compared to 2000, inpatient charges increased significantly in 2001 (11%), 2002 (31%), 2003 (45%) and 2004 (73%) respectively. LOS and other race were also found to be significantly associated with log inpatient charges in this model. For each 1 day increase in LOS, inpatient charges increased on average by 166% (95% CI, 159–173) after adjusting for other factors. Admissions that belonged to the other race category had a 19% (95% CI, 3.3–37.5) mean increase in inpatient charges compared to whites. Inpatient charges were significantly lower among blacks (-11%, 95% CI, -16.8,-6.4) and Asians (-11%, 95% CI, -17.8,-4.5) compared to whites. Males had a 10% (95% CI, 4.6–15.9) increase in inpatient charges compared to females in this model. The results of the multivariable model generated a R^2 ^of 0.7473, showing that our model accounted for 74.73% of the variance in log inpatient charges.

## Discussion

This study found that mean inflation adjusted inpatient charges for HIV/AIDS patients in Rhode Island increased significantly from 2000 to 2004, after adjusting for LOS, gender, age, race, and point of entry of hospitalization. This increase in inpatient charges followed a significantly increasing linear trend from 2000–2004. These results indicate that HIV/AIDS charges are increasing at a faster than inflation. The variable LOS helped explain most of the variance in HIV/AIDS inpatient charges. Males had significantly higher inpatient charges than females in both multivariable models. Race was also a significant predictor of inpatient charges in the multivariable model that included LOS. However, this result was likely due to the few observations in the other race category. Some admissions in the other race category had higher than expected inpatient charges most likely enabling this finding to occur.

Our findings are in contrast to the findings of studies conducted in the late 1990's, when HAART treatment led to a decrease in inpatient HIV/AIDS charges [2,19,20]. Despite the consistent increase in inpatient charges from 2000–2004, LOS was only significantly higher in 2004 compared to 2001. This suggests that there are other factors that are influencing the increase in HIV/AIDS inpatient charges.

In addition to calendar year and LOS, race, and gender influenced inpatient HIV/AIDS charges. Contrary to other studies, we found that blacks and Asians had significantly lower inpatient charges than whites[8,21]. Also, contrary to Gebo et al. this study did not find that females had significantly higher inpatient charges than men[8].

This study, like most studies, has limitations. The data we examined cannot account for multiple admissions. It is likely that there were multiple admissions by a single patient. Secondly, Rhode Island Hospital Discharge Data does not contain information on other key factors that are known to influence HIV/AIDS charges. For example, we are unable to determine the proportion of the HIV/AIDS charges that were attributable to medications, co-morbidities, physician charges, and other factors that may influence charges. We were unable to determine how inpatient charges differed by insurance status. This variable was excluded from the analyses based on the fact that more than 25% of the sample had missing insurance information. Another potential limitation is that a proportion of the uninsured HIV/AIDS admissions that were admitted may have decided to not receive hospital care due to lack of finances and thus they would not have the normal LOS and inpatient charges as others. Also, we are unable to attribute what percentage of these inpatient charges were due to other conditions that patients may have been treated for in addition to HIV/AIDS. Lastly, our results are not generalizable beyond Rhode Island. Based on this lack of generalizability we are unable to make inferences for the entire US based on our results.

Despite these limitations our study has important implications for uninsured, as well as insured HIV/AIDS patients whose health care plan does not cover HIV/AIDS treatment adequately. As stated earlier a large portion of HIV/AIDS patients in the US are uninsured. Many of these individuals are of low SES and cannot afford to purchase an adequate health care plan. The increase in HIV/AIDS inpatient charges makes it more difficult for many of these individuals to pay for their treatment, resulting in lack of treatment options which can negatively impact morbidity and mortality rates.

## Conclusion

Charges for inpatient HIV/AIDS services seem to be rising faster than inflation. Our results suggest that LOS is associated with an increase in inpatient charges from 2000–2004 in Rhode Island however the level of LOS does not seem to fully account for this increase. Other factors seem to also be influencing this increase in inpatient charges over time. It is possible that charges for medical tests and procedures are increasing however we are unable to examine these variables due to the limitations of our data. Future research should focus on obtaining informative clinical data on these medical factors in order to determine their influence on inpatient charges over time. Also, it is essential to examine if these findings are consistent in the other parts of the US.

## Competing interests

The authors declare that they have no competing interests.

## Authors' contributions

All authors provided an intellectual contribution to the study. Study design: KEB, DNP; data analysis: KEB; manuscript preparation: KEB, DNP, AF; literature review: KEB, DNP; discussion: KEB, DNP, AF.

## Pre-publication history

The pre-publication history for this paper can be accessed here:


